# The CYCLIN-A CYCA1;2/TAM Is Required for the Meiosis I to Meiosis II Transition and Cooperates with OSD1 for the Prophase to First Meiotic Division Transition

**DOI:** 10.1371/journal.pgen.1000989

**Published:** 2010-06-17

**Authors:** Isabelle d'Erfurth, Laurence Cromer, Sylvie Jolivet, Chloé Girard, Christine Horlow, Yujin Sun, Jennifer P. C. To, Luke E. Berchowitz, Gregory P. Copenhaver, Raphael Mercier

**Affiliations:** 1 Institut Jean-Pierre Bourgin, UMR1318 INRA-AgroParisTech, Versailles, France; 2 Department of Biology and the Carolina Center for Genome Sciences, The University of North Carolina at Chapel Hill, Chapel Hill, North Carolina, United States of America; 3 Lineberger Comprehensive Cancer Center, The University of North Carolina School of Medicine, Chapel Hill, North Carolina, United States of America; Stanford University School of Medicine, United States of America

## Abstract

Meiosis halves the chromosome number because its two divisions follow a single round of DNA replication. This process involves two cell transitions, the transition from prophase to the first meiotic division (meiosis I) and the unique meiosis I to meiosis II transition. We show here that the A-type cyclin CYCA1;2/TAM plays a major role in both transitions in *Arabidopsis*. A series of *tam* mutants failed to enter meiosis II and thus produced diploid spores and functional diploid gametes. These diploid gametes had a recombined genotype produced through the single meiosis I division. In addition, by combining the *tam-2* mutation with *AtSpo11-1* and *Atrec8*, we obtained plants producing diploid gametes through a mitotic-like division that were genetically identical to their parents. Thus *tam* alleles displayed phenotypes very similar to that of the previously described *osd1* mutant. Combining *tam* and *osd1* mutations leads to a failure in the prophase to meiosis I transition during male meiosis and to the production of tetraploid spores and gametes. This suggests that TAM and OSD1 are involved in the control of both meiotic transitions.

## Author Summary

In the life cycle of sexual organisms, a specialized cell division—meiosis—reduces the number of chromosomes from two sets (2n, diploid) to one set (n, haploid), while fertilization restores the original chromosome number. Meiosis reduces ploidy because it consists of two divisions following a single DNA replication. In this study, we identified genes that control the entry into the first and the second meiotic division in the model plant *Arabidopsis thaliana*. Plants lacking the *CYCA1;2* gene execute a single division during meiosis producing functional diploid gametes and polyploid plants in the next generation. By combining this mutation with two others that affect key meiotic processes, we generated plants that produce diploid gametes through a mitotic-like division that are genetically identical to their parents. Furthermore, plants lacking *CYCA1;2* and another previously described gene (*OSD1*) undergo no divisions during male meiosis, producing tetraploid pollen grains.

## Introduction

Meiosis is a central feature in the reproductive program of all sexually reproducing eukaryotes. The process of meiosis involves two rounds of chromosome segregation that follow a single round of chromosome duplication leading to the production of haploid gametes. Meiosis differs from mitosis, somatic cell division, in a number of ways. In meiosis:

- Homologous chromosomes pair and closely associate along a proteinaceous structure called the synaptonemal complex (SC). This process culminates at a substage of prophase called pachytene.
- Crossovers occur between homologs during prophase.
- Homologous chromosomes separate at anaphase of the first division. To ensure accurate chromosome segregation at anaphase I each homolog must remain connected to the other through metaphase I. Since the SC disappears before the end of prophase, it cannot ensure linkage of homologs at metaphase I. This connection is maintained until anaphase I by chiasmata, the cytological manifestation of crossovers.
- Meiosis has a second division with no intervening DNA synthesis. Sister chromatids, resulting from the meiotic S phase, remain associated until metaphase II and are separated from each other at anaphase II, leading to the production of four haploid spores [1], [2].

To generate haploid spores the meiocyte must enter meiosis I, pass through the meiosis I to meiosis II transition and exit meiosis II. Errors in these transitions, are not uncommon and may lead to parthenogenesis or teratoma formation, or to the production of gametes with the somatic number of chromosomes (2n gametes) [3], [4]. The formation of 2n gametes is thought to be an important mechanism for generating polyploids. Polyploidy has played a key role in the evolution of many fungal, plant, invertebrate and vertebrate lineages, and is particularly frequent in plants [5], [6]. 2n gametes are also an important tool for plant breeding [7].

Cyclins and cyclin-dependent kinases (Cdk) form complexes that are essential for progression through both the mitotic and meiotic cell cycles. The transition from meiosis I to meiosis II requires a fine balance in Cyclin–Cdk activity: it must be sufficiently low to exit meiosis I but must nonetheless be maintained at a level sufficiently high to suppress DNA replication and promote entry into meiosis II [8], [9]. Precisely how the mitotic machinery is modified for the purpose of meiosis is not fully understood. Most of the knowledge currently available originates from studies carried out in unicellular fungi, *Xenopus laevis* or mouse oocyte systems.

In oocytes, entry into both meiosis I and meiosis II is driven by Cdc2/Cyclin B complexes (the molecular components of the maturation promoting factor, MPF) [10]. The rate of Cyclin B synthesis and degradation by the APC (anaphase promoting complex) determines the timing of the transitions occurring during meiosis [10], [11]. At the end of meiosis I, Cyclin B is only partially degraded [12] and the residual, low level of Cdc2/CyclinB activity is essential for entry into meiosis II [13]. This partial Cyclin B degradation is fine-tuned by the Erp1/Emi2 APC inhibitor [14]–[16]. In *Schizosaccharomyces pombe*, Mes1 is a key player in the meiosis I to meiosis II transition. Like Erp1/Emi2, the Mes1 protein partially inhibits cyclin degradation by the anaphase promoting complex (APC), thereby allowing entry into meiosis II [17], [18]. In *Saccharomyces cerevisiae*, the simultaneous deletion of two of the six B-type cyclins results in a single reductional division during meiosis, with the production of two-spored asci (dyads), suggesting that specialist cyclins may be responsible for mediating the meiosis I to meiosis II transition [19]–[21].

Very little is known about control of the meiotic cell cycle in plants. In maize, the *elongate* mutant produces diploid female gametes because it is unable to undergo female meiosis II, but the corresponding gene has not been identified [22]. The only gene involved in the meiosis I to meiosis II transition isolated to date is the *Arabidopsis OSD1* (*OMISSION OF SECOND DIVISION*) gene, the molecular function of which remains unknown. As *osd1* mutants fail to enter the second division in both male and female meiosis, functional 2n gametes and tetraploid progeny are produced [23]. To look for other regulators of meiotic progression, we focused our attention on cyclins. The *Arabidopsis thaliana* genome contains 10 A-type and 11 B-type cyclins but functions in the cell cycle have been identified for only a few of these molecules, probably because redundancy attenuates the effects of mutations in single cyclin genes [24], [25]. A noticeable exception to this rule is *CYCA1;2*, also known as *TAM* (*TARDY ASYNCHRONOUS MEIOSIS*). In the *tam-1* mutant, a single substitution of an amino acid (Thr283-Ile) slows cell cycle progression during male meiosis [26], [27]. Here, we revisit the function of *CYCA1;2/TAM* by isolating a series of alleles, including null alleles, and show that *CYCA1;2/TAM* is crucial for the meiosis I to meiosis II transition. The *tam* mutants fail to enter meiosis II, leading to the production of dyads of spores and diploid gametes that have experienced genetic exchange. This demonstrates that the meiosis I to meiosis II transition in plants involves a non redundant Cyclin-cdk activity. Furthermore, combining *tam* and *osd1* mutations leads to a failure of the transition from prophase to the first meiotic division (meiosis I) during male meiosis and to the production of single-spore meiotic products and tetraploid pollen grains. This shows that CYCA1;2/TAM and OSD1 are also involved in the prophase to meiosis I transition. The implications of these results for the control of meiotic transitions are discussed.

In addition, taking advantage of a genetic strategy we used previously to investigate *OSD1* function [23], we combined *tam* mutants with mutations that affect homologous recombination (*Atspo11-1*) and chromosome segregation (*Atrec8*), essentially converting meiosis into a mitotic-like division. The *tam/Atspo11-1/Atrec8* line, called *MiMe*-2, produces diploid gametes that are genetically identical to their parents, mimicking apomeiosis, a key element of apomixis or asexual reproduction through seeds. However, *MiMe-2* also resulted in the production of diploid and aneuploid gametes, probably due to the lower penetrance of *tam* mutations compared to *osd1* mutations.

## Results

### The *tam* Mutants Produce Diploid Gametes

**Figure 1:**
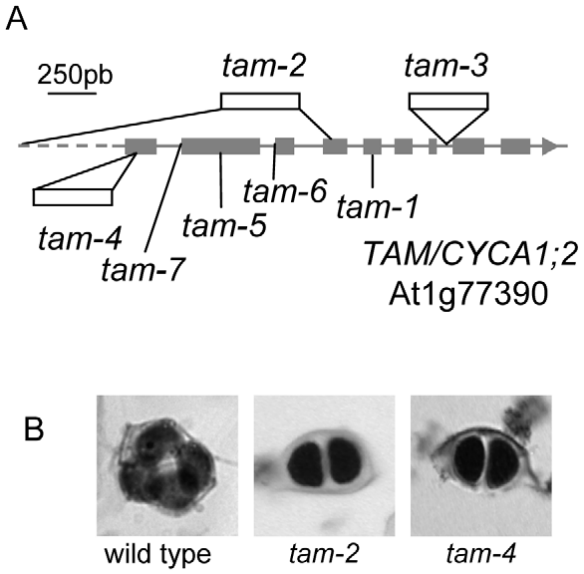
The *tam* mutations. (A) The *CYCA1;2*/*TAM* gene contains nine exons. The positions of the three insertions in the *tam-2*, *tam-3* and *tam-4* mutants are indicated as are the three point mutations in *tam-5*, *tam-6* and *tam-7*. The *tam-2* insertion is accompanied by a deletion. (B) Male meiotic products stained with toluidine blue: a tetrad from the wild-type and dyads from the *tam-2* and *tam-4* mutants.

The *Arabidopsis TAM* (Tardy Asynchronous Meiosis) gene has been implicated in the control of meiotic progression [26], [27]. The *tam-1* mutant displays slower cell cycle progression during male meiosis, although it does eventually, like wild type, produce tetrads. However, previous to this study, only one mutant allele with a point mutation leading to a single amino-acid substitution in the protein had been studied. As this point mutation is temperature-sensitive and probably corresponds to a hypomorphic allele, we revisited the role of the *TAM* gene by isolating and characterizing three independent insertion mutants from public mutant collections and three additional point mutations (Figure 1A and Figure S1). The *tam-2* (sail_505_C06 [28], [29]) T-DNA insertion is in the fourth exon (ATG+1130 pb) and is accompanied by a large deletion (corresponding to half of the *TAM* coding sequence, the entire *TAM* promoter region and the first 52 bp of the next gene, At1 g77400), the *tam-3* (SALK_080686 [30]) T-DNA insertion is in the seventh intron (ATG+1690 bp) and the *tam-4* (CSHL_ET12273 [31]) Ds insertion is in the first exon (ATG+62 bp). The *tam-2* and *tam-3* mutations are in the *Columbia* (Col-0) background, whereas the *tam-4* mutation is in the *Landsberg erecta* (Ler) background (Figure 1A). Additionally, three point mutations were isolated in an ethyl methanesulfonate (EMS) chemical mutagenesis screen. The *tam-5* mutation is a G to A transition at nucleotide 378 in the coding sequence that creates a stop codon (TGG → TAG; Trp77 → stop). The *tam-6* mutation is also a G to A transition at the -1 position of the 3′ splice site between intron 2 and exon 3. The *tam-5* and *tam-6* alleles are both recessive and do not complement one another. The *tam-7* mutation is a G to A transition at position -4 of the 3′ splice site between intron 1 and exon 2. Similar splice site proximal mutations have been observed in other *Arabidopsis* mutants including *det1-1* and *det1-3* [32]. The *tam-7* allele is dominant and thus could not be used for genetic complementation of the other *tam* alleles. All three EMS *tam* alleles are in the Columbia (Col-0) background. The T-DNA alleles were used preferentially for detailed analysis.

The *tam* mutants displayed no defects during somatic development. In *Arabidopsis*, male meiosis produces a group of four spores, organized in a tetrahedron, called a tetrad (Figure 1B). Each spore gives rise to a pollen grain. In these six independent *tam* mutants, the products of male meiosis were mostly dyads of spores instead of tetrads (Figure 1B and Table 1). In addition to balanced dyads the stronger mutants also produced triads and unbalanced products together with a very small number of tetrads. Complementation tests with *tam-2*, *tam-3 and tam-4* mutants confirmed that these mutations were allelic. Unlike the previously described temperature sensitive *tam-1* mutant, these six mutants expressed the dyad phenotype at normal growing temperatures (20°C). Furthermore, the dyad stage in *tam-1* does not appear to be terminal, as meiosis always progresses to tetrad production [27], whereas the *tam-2*, *tam-3*, *tam-4, tam-5, tam-6 and tam-7* systematically produced mostly dyads. This suggests that the *tam-1* mutant presents only a delay in the progression of meiosis, whereas the other mutants do not progress beyond the dyad stage (as confirmed by pollen analysis, see below). This phenotype is reminiscent of the phenotype of the *osd1* mutant, which produces spores directly after meiosis I. Its gametes are thus diploid and its offspring polyploid. Thus, we determined ploidy levels among the offspring of diploid *tam-2* and *tam-4* mutants. In the progeny of selfed homozygous mutants, we observed tetraploids, triploids, and occasionally diploid plants (Table 2). If pollen from *tam-2* or *tam-4* mutant plants was used to fertilize a wild-type plant, almost all the resulting progeny were triploid, with only a few diploid plants identified (Table 2). If *tam-2* or *tam-4* mutant ovules were fertilized with wild-type pollen grains we isolated diploid and triploid plants (Table 2). Thus, the frequency of diploid spores, resulting in functional gametes, was high in the *tam-2* and *tam-4* mutants, for both the male (∼90%) and female (∼30%) lineages. *Tam* mutants produced only slightly fewer seeds than the corresponding wild-type lines (36±4 seeds/silique in *tam-2*; 42±4 in wild type Col-0; 43±4 in *tam-4*; 52±3 in wild type Ler), but many (>50%) of the seeds produced by *tam-2* mutants and a few (<10%) of those produced by *tam-4* mutants were shriveled. This finding was not unexpected, because *tam* mutants produce triploid seeds with an excess of the paternal genome that is associated with the production of shriveled seed, particularly in the Col-0 background [33]. The germination rate of seeds produced by *tam-2* was 63% (n = 264), suggesting that the proportion of triploid seeds may be underestimated in *tam-2* progeny.

**Table 1:** Male meiotic products in *tam* mutants.

	*tam-2*	*tam-3*	*tam-4*	*tam-5*	*tam-6*	*tam-7*
frequency of dyads	89%	87%	84%	62%	70.1	82%
Tetrads	0.3%	0.4%	5%	1.1%	1.9	15%
Triads and unbalanced products	11%	13%	11%	26%	28%	3%
number of meiotic products	224	251	182	860	755	66

**Table 2:** Ploidy of *tam*, *tam/osd1*, and *MiMe-2* progenies.

		*tam-2*	*tam-4*	*tam-2/osd1-1*	*MiMe-2*
selfed progenies	2N	2%	7%		
	3N	58%	67%	8%	
	4N	40%	25%	60%	85%
	6N frequency			32%	
	aneuploid frequency				15%
	number of plants	64	27	132	20
wt♀ x mutant ♂	2N frequency	5%	12%	-	10%
	3N frequency	95%	88%	-	83%
	aneuploid frequency			-	7%
	number of plants	85	25	-	45
mutant♀ x wt♂	2N frequency	61%	75%	1%	-
	3N frequency	39%	25%	99%	89%
	aneuploid frequency				11%
	number of plants	61	16	73	45

### The *tam* Mutants Skip the Second Division of Meiosis

**Figure 2:**
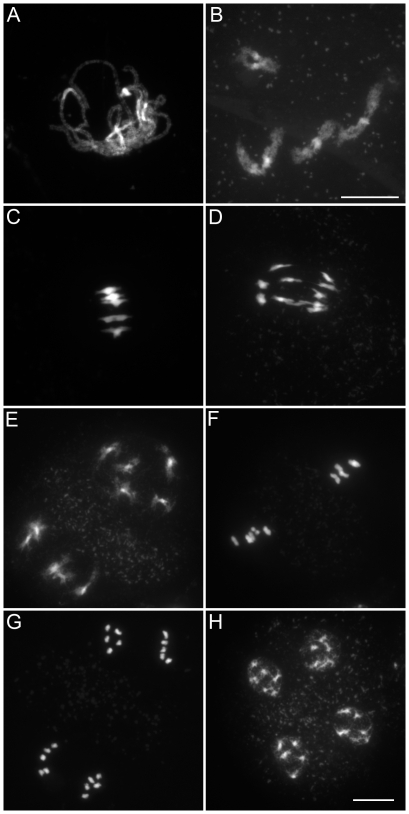
Male meiosis in wild type. Micrographs of *Arabidopsis* chromosome spreads stained with 4',6-diamidino-2-phenylindole (DAPI) at (A) Pachytene; (B) Diakinesis; (C) Metaphase I; (D) Anaphase I; (E) Telophase I; (F) Metaphase II; (G) Anaphase II; and (H) Telophase II. Scale bar = 10 µm.

**Figure 3:**
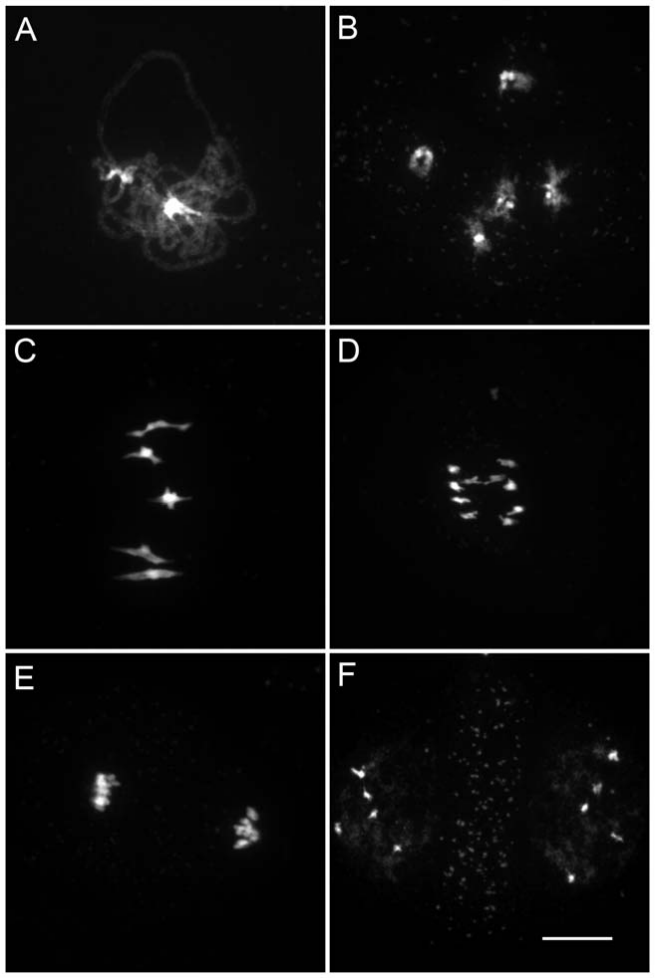
Male meiosis in the *tam-2* mutant. Micrographs of DAPI stained *Arabidopsis* chromosomes show that *tam-2* Meiosis I including (A) Pachytene; (B) Diakinesis; (C) Metaphase I; (D, E) Anaphase I; and (F) Telophase is indistinguishable from the wild type but no figures characteristic of a second division were observed. I. Scale bar = 10 µm.

**Figure 4:**
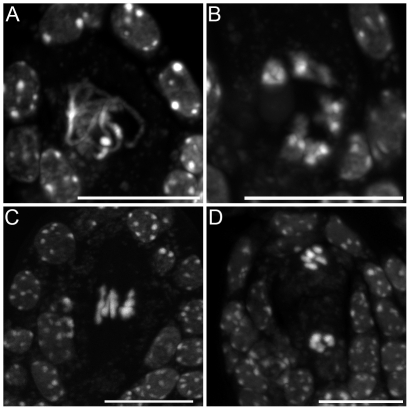
Female meiosis in wild type. Micrographs of DAPI stained *Arabidopsis* chromosomes during (A) Pachytene; (B) Diakinesis; (C) Metaphase I; and (D) Anaphase I. Scale bar = 10 µm.

**Figure 5:**
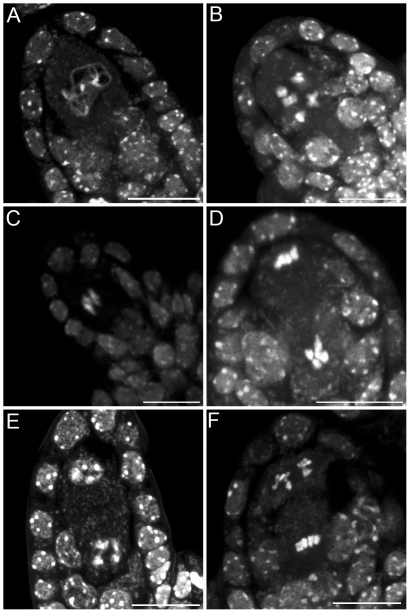
Female meiosis in the *tam-2* mutant. Micrographs of DAPI stained *Arabidopsis* chromosomes in *tam-2* show that both meiosis I (A) Pachytene; (B) Diakinesis with five bivalents; (C) Metaphase I; (D) Anaphase I; (E) Telophase I and meiosis II (F) Metaphase II and anaphase II were indistinguishable from wild type. Scale bar = 10 µm.

We characterized the mechanisms underlying dyad production in *tam* mutants, by investigating chromosome behavior during male meiosis using a meiocyte spreading technique (Figure 2 and Figure 3). In wild type, the ten chromosomes appeared as threads at leptotene, underwent synapsis at zygotene and were fully synapsed, along the SC at pachytene (Figure 2A). After the disappearance of the SC at diplotene the resulting five bivalents condensed, revealing the presence of chiasmata (Figure 2B). The bivalents organized on the metaphase I plate (Figure 2C) and homologs segregated to opposite poles at anaphase I (Figure 2D and 2E). The two sets of five homologs aligned on the two metaphase II plates (Figure 2F). The second round of segregation at anaphase II (Figure 2G) led to the formation of four sets of five chromosomes, that decondensed to form the spore nuclei (Figure 2H). Meiosis I in *tam* mutants was indistinguishable from that in the wild type. All stages of prophase were observed, including full synapsis at pachytene (Figure 3A) and chiasmata at diakinesis (Figure 3B). The bivalents observed at diakinesis, condensed and aligned on the metaphase plate (Figure 3C). We quantified the chiasma frequency by studying the shape of metaphase I bivalents, as described by [34], [35], and found no difference between *tam-2* (9±0.8 chiasmata per cell, n = 70) and the wild type (9.1±1 chiasmata per cell, n = 53). The bivalents segregated at anaphase I and decondensed at telophase I (Figure 3D–3F). However, we found no meiosis II figures (among >1000 male meiocytes for each *tam-2*, *tam-3* and *tam-4*, from prophase to spore formation), consistent with the dyad production in *tam* mutants resulting from the absence of a second meiotic division. Both typical meiosis I and meiosis II figures were observed during female meiosis (Compare Figure 4 and Figure 5), consistent with the observed production of ∼70% haploid female gametes in *tam* mutants and the notion that female diploid megaspores are also produced by skipping the second meiotic division.

**Figure 6:**
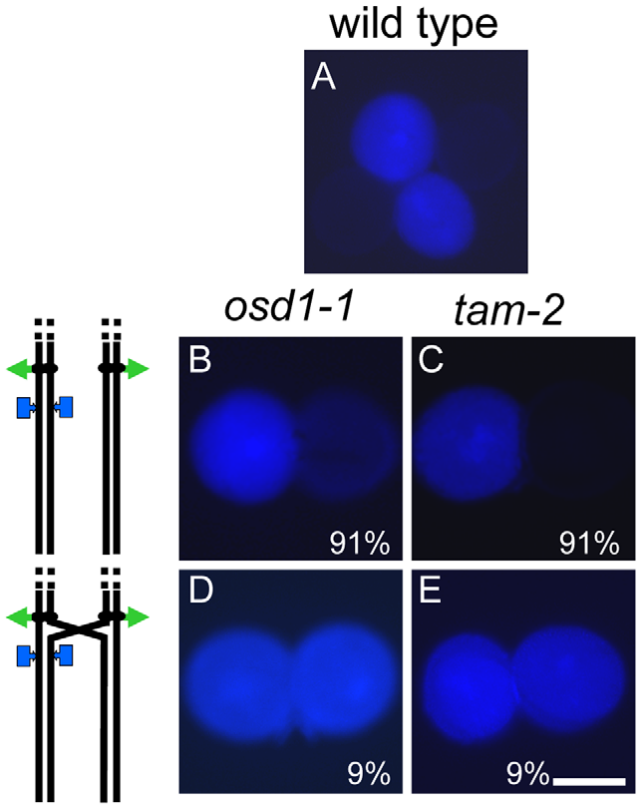
Single-locus (FTL3253) segregation patterns in pollen. A tetrad of wild-type (*qrt1-2* background) pollen (A) from a plant that was hemizygous for a transgene encoding a cyan fluorescent protein has two fluorescent and two non-fluorescent pollen grains. Dyads of pollen (B and C) from the *osd1-1* and *tam-2* mutants, respectively, having one fluorescent and one non-fluorescent pollen grain in each case. Dyads (D and E) from the *osd1-1* and *tam-2* mutants, respectively, each showing two fluorescent pollen grains (*osd1* n = 173, *tam* n = 214). Interpretations, of chromosome segregation and crossover position, are shown on the left. Scale bar = 20 µm.

**Figure 7:**
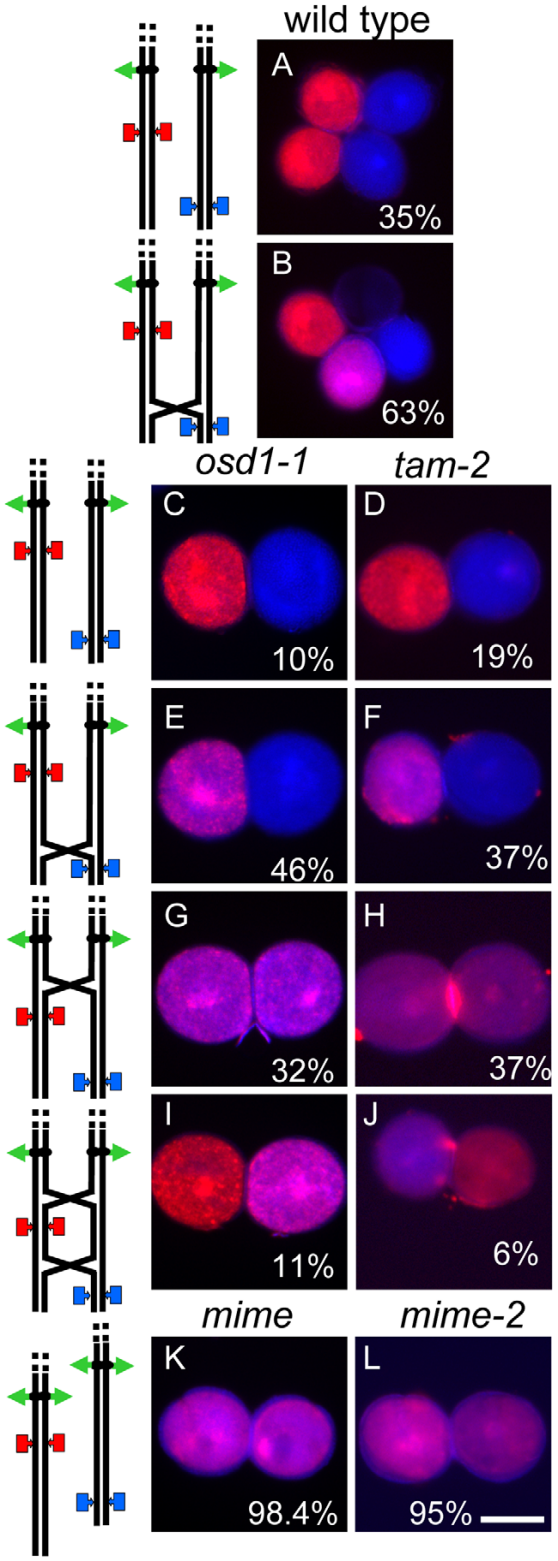
Two-locus (FTL1273 red, FTL993 blue) segregation pattern in pollen. (A) Tetrad of wild type pollen with two blue fluorescent and two red fluorescent pollen grains, indicative of an absence of recombination between the markers. A crossover between the centromere and the upper marker may or may not have occurred. (B) Tetrad of wild-type pollen with one blue fluorescent, one red fluorescent pollen, one blue and red fluorescent and one non-fluorescent pollen grain, indicative of a crossover between the two markers. (C, D) Dyads of pollen from the *osd1-1* and *tam-2* mutants, respectively, each with one blue fluorescent and one red fluorescent pollen grain. (E, F) Dyads from *osd1-1* and *tam-2* mutants, respectively, each with one blue and red fluorescent pollen grain and one blue fluorescent pollen grains. (G, H) Dyads from *osd1-1* and *tam-2* mutants, respectively, each with two blue and red fluorescent pollen grain. (I, J) Dyad in *osd1-1* and *tam-2*, respectively, with one blue and red fluorescent pollen grain and one red fluorescent pollen grain. (K, L) Dyads from *MiMe-1* and *MiMe-2* plants, respectively, each with two blue and red fluorescent pollen grains. Interpretations of chromosome segregation and crossover position, are shown on the left. (wild type n = 266, *osd1* n = 263, *tam* n = 369). Rare cases indicative of multiple crossovers are not shown. Scale bar = 20 µm.

**Figure 8:**
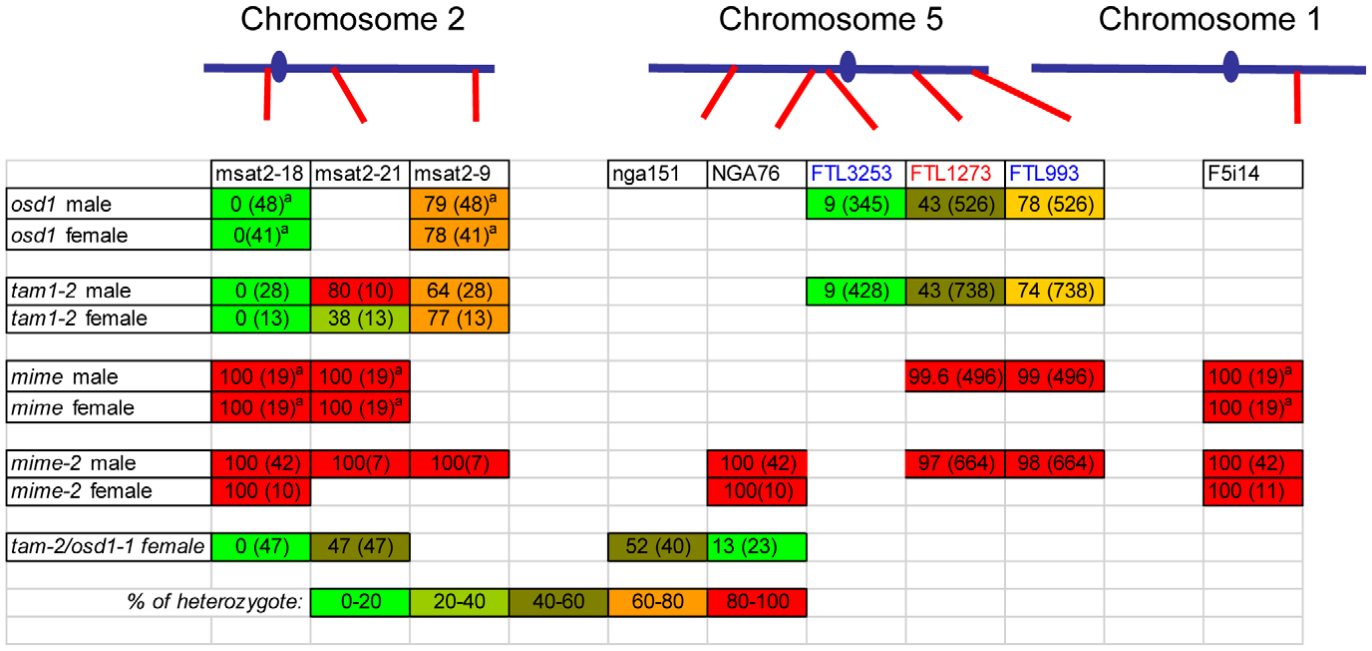
Genetic make-up of the *osd1, tam, tam/osd1*, *MiMe,* and *MiMe-2* male and female diploid gametes. Genotype of pollen grains, for the FTL loci (Figure 6 and Figure 7). In addition triploid offspring from mutant x wild-type crosses were genotyped for several molecular markers. The percentage of heterozygous gametes was determined (n is indicated into brackets). The frequency of heterozity is indicated by a color code, from 0% (green) to 100% (red). The positions of each marker (red) and the centromeres (blue) are indicated on the chromosomes. ^a^data from [23].

If diploid gametes are indeed generated by skipping the second meiotic division, any parental heterozygosity at centromeres should be lost in the *tam* diploid gametes, because sister centromeres cosegregate at division I. Conversely, we would also expect heterozygosity to increase towards telomeres, due to recombination between the locus concerned and the centromere. We tested this hypothesis in two ways, using the fluorescent tagged line (FTL) *Arabidopsis* tetrads system providing direct information about the genetic content of pollen grains (Figure 6, Figure 7, Figure 8) and by genotyping male and female *tam-2* triploid offspring for polymorphic molecular markers (Figure 8) [23], [36], [37].

The FTL system is a visual assay based on the use of reporter constructs encoding fluorescent proteins produced in the pollen of the *quartet* mutant (*qrt1-2*) [38]. In this mutant, the pollen grains from each meiosis remain physically attached. We carried out crosses to generate plants with both the *qrt1-2* and *tam-2* mutations, heterozygous for one or two reporter transgenes conferring pollen fluorescence. As controls, we used both wild type and *osd1* mutant plants. We first used an FTL transgene located close to the centromere on chromosome 5 (FTL3253 encoding AmCyan) [39]. In wild-type (*qrt1-2* background) control plants, all tetrads consisted of two fluorescent and two non-fluorescent pollen grains (Figure 6A), reflecting the segregation of the four chromatids, two of which carried the transgene (n = 120). In the *osd1-1* and *tam-2* mutants, we observed mostly dyads of pollen (Figure 6B–6E). Furthermore, in both mutants, the vast majority of dyads (91%, n = 526 and n = 738, in *osd1-1* and *tam-2* respectively) consisted of one fluorescent pollen grain and one non-fluorescent pollen grain (Figure 6B and 6C). Thus, in these dyads, one of the pollen grains contained the two sister chromatids carrying the transgene, whereas the other pollen grain inherited the other two sister chromatids. This segregation pattern is fully consistent with the absence of a second meiotic division. In a small proportion of dyads (9% in both *osd1-1* and *tam-2* mutants) both pollen grains were fluorescent, indicating recombination between the transgene and the centromere (Figure 6D and 6E). We then carried out the same experiment with two linked FTL transgenes located at some distance from the centromere on chromosome 5 (FTL1273 DsRed2, FTL993 ECFP) [39]. In the wild type (*qrt1-2* background), we observed tetrads without (Figure 7A) and with (Figure 7B) recombination of the markers as described in a previous study [36]. The frequency of recombination between the two markers, measured as the percentage of non-parental chromatids deduced from the fluorescence distribution, was 32%. In both the *osd1-1* and *tam-2* mutants, we observed segregation patterns in the dyads reflecting crossing over between the two markers (29% (n = 262) and 22% (n = 367), respectively) and between the markers and the centromere (22% and 22%, respectively) (Figure 7C–7J). The frequency of heterozygosity at the FTL marker loci in diploid pollen grains was determined (Figure 8). The frequency of heterozygosity was low next to the centromere and increased with distance from the centromere. All these findings are consistent with the *tam* and *osd1* diploid pollen grains resulting from a single first division of meiosis that includes recombination, with no second division [23].

For confirmation of this genetic analysis, we genotyped triploid offspring generated from male and female gametes from the *tam-2* mutant. We first introduced genetic polymorphisms into *tam-2* (Col-0 background) by crossing this mutant to the No-0 accession. In the F2 generation, we used PCR to select plants homozygous for the *tam-2* mutation but heterozygous for a series of microsatellite markers. These plants were crossed, as male or female parents, with plants from a third genetic background (Landsberg erecta, Ler). Genotyping of the resultant triploid progeny for the trimorphic molecular markers revealed the genetic make-up of the 2n gametes produced by the mutant. The Ler allele was present in all the plants (brought by the wild type Ler haploid gamete), while the presence/absence of the Col-0/No-0 allele in the triploids corresponds to the genotype of the 2N mutant gametes. All the diploid gametes tested had the predicted genetic characteristics, similar to those of the diploid gametes produced by the *osd1* mutant (Figure 8). The markers were homozygous at the centromere, but displayed segregation at other loci, due to recombination. These results confirmed that the absence of a second meiotic division was indeed responsible for the production of both male and female 2n gametes in *tam-2*.

### Turning Meiosis into Mitosis

**Figure 9:**
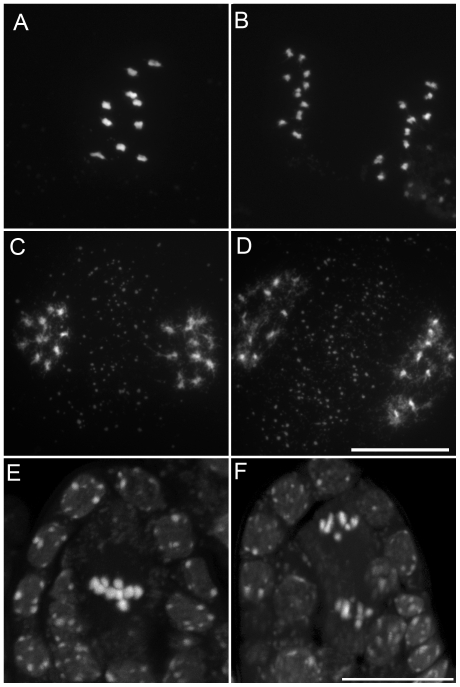
Mitosis-like division instead of meiosis, in *MiMe-2* plants. (A–D) Male Meiosis. (A) Metaphase I. (B) Anaphase I. (C, D) Telophase I. (E–F) Female meiosis. (E) Metaphase I. (F) Anaphase I. Scale bar = 10 µm.

Our *tam* alleles displayed phenotypes very similar to that of the *osd1* mutant, with no second meiotic division, leading to the production of viable diploid male and female gametes. Thus, *tam* diploid gametes differ from apomeiotic (mitosis-like) gametes in that they are genetically different from the mother plant [40], [41]. We have previously shown that, by combining the *osd1* mutation with the *Atspo11-1* mutant, which eliminates recombination, and the *Atrec8* mutation, which modifies chromatid segregation, it is possible to convert meiosis into a mitosis-like division (apomeiosis). We called this triple mutant *MiMe* for “mitosis instead of meiosis” [23]. We investigated whether *tam* mutation could replace the *osd1* mutation to convert meiosis into mitosis, by constructing the *tam-2/Atspo11-1/Atrec8* triple mutant, which we named *MiMe-2*. During male meiosis, *MiMe-2* plants mostly generated dyads (91.2% 446/489), with only a small number of unbalanced products. Observations of chromosome behavior during male and female meiosis showed that the division process resembled mitosis: 10 univalents aligned on the metaphase plate, with the separation of sister chromatids at anaphase (Figure 9). The selfed progeny of *MiMe-2* plants was mostly tetraploid, with some aneuploids (Table 2). Backcrossing *MiMe-2* plants, as the female parent, onto wild-type plants resulted mostly in triploids with a few aneuploid plants (Table 2), whereas backcrossing *MiMe* plants, as the male parent, onto the wild type, generated a mixture of mostly triploid plants, diploid and aneuploid plants (Table 2). Thus, the mitosis-like division observed in *MiMe-2* plants gives rise to functional diploid gametes, together with small numbers of haploid and aneuploid gametes, in both the male and female lineages. *MiMe-2* plants also displayed lower levels of fertility than either the wild-type or the *tam-2* mutant (wild type: 42±4 seeds/fruit; *tam-2*: 37±4, *MiMe2*: 20±3). This finding was not unexpected, as the *tam* mutation does not display full penetrance. In meiocytes lacking *Atrec8* and *Atspo11* that undergo a second division, this division is unbalanced, probably leading to the production of aneuploid gametes in *MiMe-2* plants [42]. We analyzed the genetic content of the *MiMe-2* diploid gametes, using both the FTL lines and molecular markers (Figure 7K and 7L and Figure 8). We introduced the same FTL transgenes as described above (FTL1273 DsRed2, FTL993 ECFP) into *MiMe* and *MiMe-2* plants. Almost all the pollen grains of both genotypes displayed both types of fluorescence (Figure 7K and 7L; 98% and 95%, respectively), indicating that they had inherited both transgenes, and confirming the occurrence of a mitosis-like division, rather than meiosis, in both lines. The few cases in which the two pollen grains were not expressing both fluorescent proteins may be explained by chromosome missegregation or occasional extinction of the transgenes. In addition, all the diploid *MiMe-2* gametes (male and female) systematically retained heterozygosity for each genetic marker tested (Figure 8). They were thus genetically identical to the mother plant. These results confirm that *MiMe-2* plants undergo a mitosis-like division instead of a normal meiotic division, generating gametes genetically identical to the parent plant, but at a lower regularity than *MiMe* plants [23].

### *tam/osd1* Double Mutant Skip Division II at Female Meiosis and Skip Both Divisions at Male Meiosis

**Figure 10:**
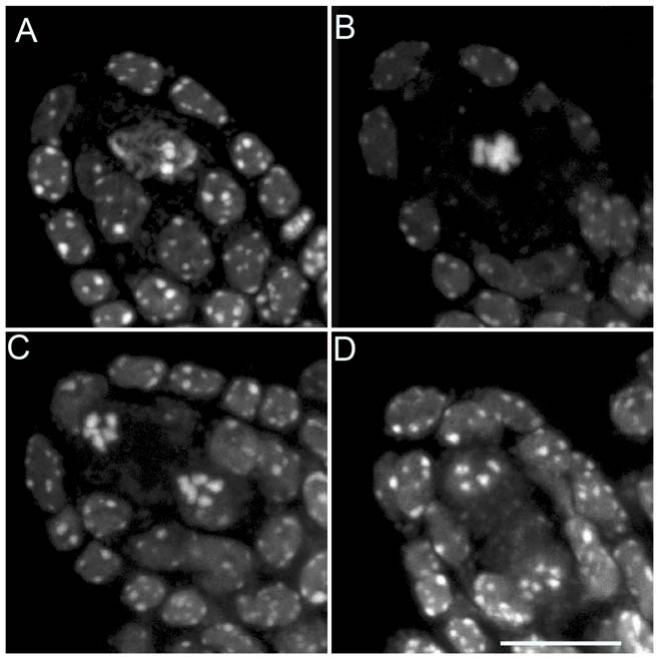
Female meiosis in the *tam-2/osd1-1* double mutant. Meiosis I is indistinguishable from the wild type but no figures characteristic of a second division were observed. (A) Pachytene. (B) Metaphase I. (C) Anaphase I. (D) Telophase I. Scale bar = 10 µm.

Our *tam* alleles displayed phenotypes very similar to that of the *osd1* mutant, with no second meiotic division, leading to the production of viable diploid male and female gametes. We then combined *tam-2* and *osd1-1* mutations. The double mutant was almost sterile, producing very few seeds by selfing. Reciprocal crosses with wild type revealed that *tam-2/osd1-1* double mutant was female fertile but male sterile. If *tam-2/osd1-1* mutant ovules were fertilized with wild-type pollen grains we isolated almost exclusively triploid plants (Table 2). Observation of female meiosis in the double mutant revealed normal meiosis I but an absence of meiosis II (Figure 10). The genetic analysis of the female gametes, performed as above, showed that all the diploid ovules tested had the predicted genetic characteristics for an absence of second meiotic division (Figure 8). These results show that *tam-2/osd1-1* double mutants display the same female meiosis phenotype as single mutants, with female meiocytes failing to enter meiosis II, leading to the production of 2n ovules.

**Figure 11:**
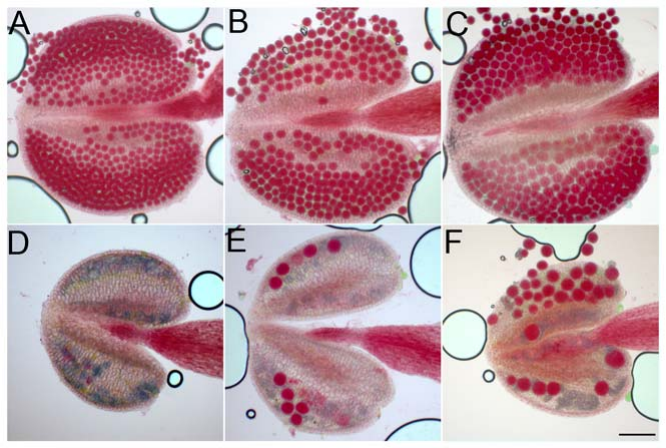
Pollen grains in wild type, *osd1*, *tam* and *tam/osd1*. Anthers were stained as described by Alexander [43]. (A) Wild type. (B) *tam-2* (C) *osd1-1*. (D–F) *tam-2/osd1*. Scale bar = 100 µm.

**Figure 12:**
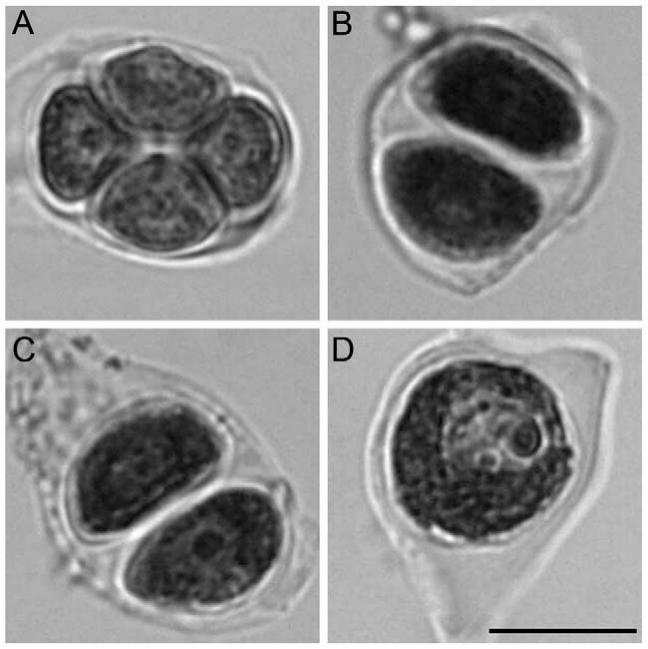
Male meiotic products stained with toluidine blue. (A) Wild type. (B) *tam-2*. (C) *osd1-2*. (D) *tam-2/osd1-1*. Scale bar = 10 µm.

**Figure 13:**
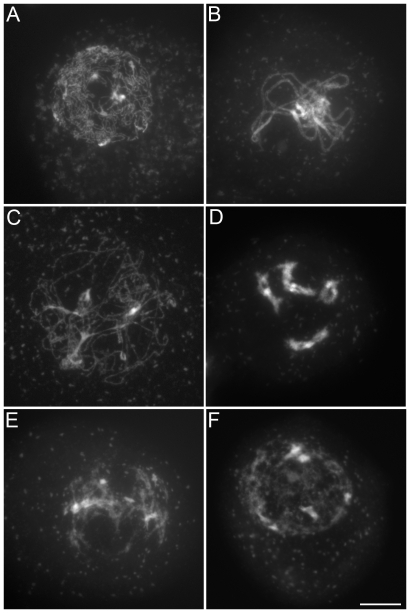
Male meiosis in *tam-2/osd1-1*. Prophase was indistinguishable from wild type. (A) Leptotene. (B) Pachytene. (C) Diplotene. (D) Diakinesis. (E) Spore formation. (F) Spore. Scale bar = 10 µm.

We investigated the origin of the male sterility phenotype observed in *tam-2/osd1-1* double mutant plants, by assessing pollen viability [43]. Figure 11A shows a wild-type anther treated with Alexander stain which produces a red pigment in viable pollen. Anthers of *tam-2* and *osd1-1* single mutants contained viable pollen grains, although less numerous and slightly bigger than wild type (Figure 11B and 11C). In contrast, *tam-2/osd1-1* anther contained very few pollen grains (9±11) with variable size (Figure 11D–11F). In wild type, meiosis produces four spores (Figure 12A), whereas both *tam* and *osd1* mutants produce dyads of spores (Figure 12B and 12C). In contrast, observation of male meiotic products in *tam-2/osd1-1* revealed only “monads”, with a single-spore product (Figure 12D, n = 498). We then investigated the behavior of male meiotic chromosomes in *tam-2/osd1-1* mutants (Figure 13). Prophase was indistinguishable from wild type: the ten chromosomes appeared as threads at leptotene, underwent synapsis at zygotene and were fully synapsed at pachytene. After the disappearance of the SC at diplotene, the resulting five bivalents condensed, revealing the presence of chiasmata. However, we observed spores with a single nucleus (Figure 13E and 13F), consistent with the observation of monads. Furthermore, only two figures typical of metaphase/anaphase I and no figures of telophase I or meiosis II were found among >1600 meiocytes. This suggests that most male meiocytes skip both meiosis I and II and produce spores directly after replication and prophase, without chromosome segregation. An expected consequence of such a defect is the production of 4n pollen grains. We did not succeed in crossing *tam-2/osd1-1* mutants as male but we determined ploidy levels among the seeds produced by selfing and found a large proportion of 6n plants in the progeny of 2n plants (Table 2). As crosses with wild type showed that *tam-2/osd1-1* produce 2n ovules, the occurrence of 6n plants strongly suggests that 4n pollen grains are produced, in accordance with the skipping of both rounds of segregation at male meiosis in *tam-2/osd1-1*. A large proportion of 4n plants is also found in the selfed progeny showing that *tam-2/osd1-1* also produces 2n pollen grains. These 2n pollen grains likely originate from the few meiocytes that enter meiosis I and produces 2n spores, that later outcompete 4n pollen.

## Discussion

### A Cyclin A Mediates the Meiosis I to Meiosis II Transition

We show here that one of the ten known type A cyclins in *Arabidopsis*, CYCA1;2/TAM, is required for the transition between meiosis I and meiosis II. No phenotype has been reported for mutants lacking any of the other CYCA, probably due to the high level of redundancy [24]. By contrast, none of the other cyclins were able to compensate for CYCA1;2 in the meiosis I to meiosis II transition. CYCA1;2 may have a specialist function or pattern of expression, required for this transition, that cannot be supplied by any of the other cyclins from *Arabidopsis*. Alternatively, the lack of CYCA1;2 may decrease generic cyclin/CDK complex activity, causing the meiosis I-meiosis II transition to fail.

### CYCA1;2 and OSD1 Are Involved in the Prophase to Meiosis I Transition at Male Meiosis

Both *cyca1;2/tam* and the previously described *osd1* mutants fail to enter meiosis II and produce spores after meiosis I. Remarkably, male meiocytes lacking both *OSD1* and *CYCA1;2/TAM* genes fail to enter meiotic division I, producing spores directly after prophase. This shows that in addition to their crucial function in driving meiosis I to meiosis II transition, these two genes are involved in the prophase to meiosis I transition. This suggests that they both contribute to an activity promoting entry into meiosis I and entry into meiosis II, most likely a CDK activity. The molecular function of OSD1 is currently unknown, however, it has been proposed by analogy to Erp1/Emi2 and Mes1 that it may inhibit APC activity, thus promoting CDK activity [23]. TAM/CYCA1;2 being a cyclin, may directly promote CDK activity. We believe that the meiosis I to meiosis II transition is easily disturbed, because fine regulation of the levels of cyclin/CDK activity is required to ensure both exit from meiosis I and entry into meiosis II [8]. Thus a moderate decrease of CDK activity in *osd1* and *cyca1;2/tam* single mutants may cause failure to enter meiosis II without impairing the prophase to meiosis I transition. In contrast the coincident depletion of OSD1 and CYCA1;2/TAM, may further decrease CDK activity, impairing entry into meiosis I. Unfortunately the direct measurement of CDK activity during meiosis in *Arabidopsis* is currently not possible. The combination of *osd1* and/or *cyca1;2/tam* mutants with other mutants affecting CDK activity may help to test this model.

In *S. cerevisiae*, the simultaneous deletion of two (out of six possible) B-type cyclins (Clb1 and Clb3 or Clb1 and Clb4) leads to a failure to enter meiosis II [20], [21]. However, although Clb3 activity is specific to meiosis II, this is not the case for Clb1 and Clb4 [19], and the *Clb1 Clb3 Clb4* triple mutant barely sporulates, suggesting that all three proteins have functions in meiosis progression other than the meiosis I-meiosis II transition, similar to OSD1 and CYCA1;2/TAM.

### Different Control of Male and Female Meiosis

In the *osd1/tam* double mutant, male meiocytes fail to enter meiosis I after prophase, whereas female meiocytes proceed to meiosis I and fail to enter meiosis II revealing a striking difference in the control of male and female meiosis progression. We suggest that other cyclins may partly compensate for the absence of CYCA1;2, during female meiosis, and that the meiosis I to meiosis II transition may be driven by different mixtures of cyclins in male and female meiosis. This possibility is supported by the *cyca1;2/tam* single mutants being weakly affected in the female lineage, compared to the male lineage and to *osd1* male and female lineage. An analysis of the effects on meiosis of the depletion of other cyclins, either alone or together with TAM/CYCA1;2, is required to test this hypothesis.

Cyclin A proteins specific to male meiosis have already been described in mammals. The mouse and human genomes each contain two different A-type cyclins. One of these cyclins, Cyclin A1, is restricted to the germ line whereas Cyclin A2 is ubiquitously expressed [44]–[47]. The loss of Cyclin A1 function has no effect on viability and results in male sterility, with male meiosis progressing to the late prophase and then leading to apoptosis; it has no effect on female fertility [48]. Control of male and female meiosis by a different set of cyclins may thus be a general phenomenon.

### Plant Cyclins and Meiosis

The *Arabidopsis* genome encodes 50 cyclins or putative cyclins [25]. Two of these cyclins, SDS and CYCA1;2/TAM, have been directly implicated in meiosis. CYCA1;2/TAM is a type A cyclin involved in cell cycle progression [27] [25 and this study], whereas SDS forms an outgroup, and plays a specific role in recombination with no evidence for any role in cell cycle progression [49], [50]. SDS is required to bias crossovers between homologous chromosome at meiosis rather than between sister chromatid, as in mitosis [50]. A viable hypomorphic mutant of CDKA displays meiotic defects potentially corresponding to a combination of the *sds* and *tam* defects [51] (e.g. recombination and progression defects), consistent with both SDS and TAM/CYCA1;2 being involved in CDKA activation. In wheat, *Cdc2/CDKA* genes are essential components of the *Ph1* locus, which is responsible for preventing recombination between homeologous chromosomes [52]. This suggests that cyclin/CDK activity may finely regulate various meiotic events.

### Relevance for Apomixis

Apomixis, or asexual clonal reproduction through seeds, has great potential for agricultural applications [40], [41]. It can be separated into three developmental components: an absence or alteration of meiosis (apomeiosis), the fertilization-independent development of the embryo from the egg cell (parthenogenesis), and the initiation of endosperm development with or without fertilization [40], [41], [53]. We recently showed that fully penetrant apomeiosis can be induced in *Arabidopsis*, when meiosis is replaced by a mitosis-like division in the *MiMe* genotype, which combines mutations in three genes, *AtSPO11-1*, to eliminate recombination, *AtREC8*, to ensure the separation of sister chromatids rather than homologues and *OSD1* to abolish the second division [23]. We have now identified a second gene, *CYCA1;2/TAM,* the mutation of which abolishes the second division of meiosis. The *MiMe-2* genotype, which combines the *Atspo11-1*, *AtRec8* and a newly described *tam* mutation, gives rise to the same phenotype as the *MiMe* genotype, with the conversion of meiosis into a mitosis-like division. Thus, apomeiosis can be engineered by combining various mutations. However, as *tam* mutations have a lower penetrance than *osd1* mutations, *MiMe-2* plants produce apomeiotic gametes less frequently than *MiMe* plants. Thus, *osd1* mutations may be more suitable than *tam* mutations for agricultural applications, if the results obtained in *Arabidopsis* can be extrapolated to crop plants. In addition to apomeiosis in *MiMe* or *MiMe-2* plants, apomixis will required the introduction of parthenogenesis and autonomous endosperm formation.

## Materials and Methods

### Growth Conditions and Genotyping

*Arabidopsis* plants were cultivated as previously described [54]. For cytometry experiments, *Arabidopsis* plants were cultivated on *Arabidopsis* medium [55], at 21°C, under a 16-h to 18-h photoperiod and 70% relative humidity. The *tam-2* (Sail_505-C06 Columbia accession), *tam-3* (Salk_080686 Columbia accession), *tam-4* (CSHL_Et12273 Landsberg *erecta* accession), *tam-5*, *tam-6* and *tam-7* mutants were genotyped by PCR. For *tam-2*, *tam-3* and *tam-4* two primer pairs were used. The first pair is specific to the wild-type allele and the second pair is specific to the left border of the inserted sequence. *tam-2*: N874380U (5′ GACTTGATGGATCCACAGC 3′) & N874380L (5′ CAGAAATCCTCCACTTGCG 3′); N874380L & LB3Sail (5′ TAGCATCTGAATTTCATAACCAATCTCGATACAC 3′). *tam-3*: N580686U (5′ GAAGAGTATAGGCTTTCGCCC 3′) & N580686L (5′ TGCAACCACATCAGATGAGAG 3′); N580686L & LBSalk2 (5′ GCTTTCTTCCCTTCCTTTCTC 3′). *tam-4*: ET12273R (5′ TAATGGGACCCACTGATGATC 3′) & ET12273L (5′ ACCTCAGATACACGCAAATGC 3′); ET12273L & Ds5-1 (5′ GAAACGGTCGGGAAACTAGCTCTAC 3′). *tam-5* F2P24.10_3 (5′ CATCGCTTTGGAGCAATTCGGTGT) & F2P24.10_555 (ACCAAAACCTGCTTTATCTCGCAATT. tam-6 F2P24.10_cf (CACCATGTCTTCTTCGTCGAGAAATCTATCTCA) & F2P24.10_572 (TGGCCGATCTTATTTGAACAATTCACCTC). *tam-7* F2P24.10_4 (AAGACACCGAATTGCTCCAAAGCG) & F2P24.10_yjd (ATGTAATCTAGAGCCGGTCTTTTGTTCAA). The PCR products for *tam-5*, *tam-6* and *tam-7* were designed as derived amplified polymorphic sequences (dCAPS) [56] using the Sainsbury atPRIMER webtool (http://www.atprimer.tsl.ac.uk/cgi-bin/form1.cgi). The dCAPS for *tam-5*, *tam-6* and *tam-7* are cleaved with Mfe I (125 bp versus 103 bp+22 bp), Xba I (316 bp versus 136 bp+180 bp) and Mfe I (396 bp versus 365 bp+31 bp) respectively. The cleaved amplified polymorphic sequences (CAPS) used to genotype *qrt1-2* were described by Francis *et a*l [39]. The primers used to genotype *osd1-1*, *Atspo11-1-3* and *Atrec8-3* were described in a previous study [23]. The *tam-2* and *tam-*4 T-DNA right border junction was analyzed by sequencing PCR products. For *tam-2*, the specific primers used were 77400F (5′TTGGCGAATCGTGGCGAGAA 3′) and Rb3Sail (5′TAACAATTTCACACAGGAAACAGCTATGAC3′. For *tam-4*, the specific primers used were ET12273R and Ds3-4 (5′ CCGTCCCGCAAGTTAAATATG3′).

### Fluorescent Tagged Lines

The seeds for FTL analysis were obtained from G.P. Copenhaver [36], [57]. We obtained *tam-2/qrt1-2* and FTL3253 +/− plants by selfing double heterozygous *tam-2/qrt1-2*, FTL3253 +/− plants. We obtained *osd1-1/qrt1-2* and FTL3253 +/− plants by selfing a double heterozygote *osd1-1/qrt1-2* FTL3253 +/− plants. We obtained *tam-2/spo11-1-3/rec8-3/qrt1-2* and FTL993 +/− FTL1273 +/− plants by selfing a *qrt1-2* mutant, triple heterozygous *tam-2/spo11-1-3/rec8-3* and FTL993 +/− FTL1273 +/− plant. We obtained *osd1-1/spo11-1-3/rec8-3/qrt* and FTL993 +/− FTL1273 +/− plants by crossing a *qrt1-2* mutant, triple heterozygote *osd1-1/spo11-1-3 /rec8-3* FTL993 −/− FTL1273 +/+ plant with a *qrt1-2* mutant, triple heterozygous *osd1-1/spo11-1-3/rec8-3* FTL993 +/+ FTL1273 −/− plant.

Plants of interest were selected by PCR genotyping. For each line, the first pair of primers is specific to the wild-type allele and the second pair is specific to the T-DNA insertion:

FTL3253 (AmCyan, nucleotide position 9304032 on chromosome 5): FTL3253U (5′ AACTTAGATGCCGAAGAAATG 3′) & FTL3253L (5′ GAGATTCTATACAGATTGATCC 3′);

FTL3253U & LB-TDNA_FTL (5′ GGCATGCAAGCTGATAATTC3′)

FTL993 (CFP, nucleotide position 25731311 on chromosome 5): FTL993U (5′ AGTGACAAGAATCCTAGTCG 3′) & FTL993L (5′GTCTCTACTAAGAGCTCCTC 3′); FTL993L & LB-TDNA_FTL

FTL1273 (DsRed2, nucleotide position 18164269 on chromosome 5): FTL1273U (5′ TACTTAGTCTAGGGTATACAC3′)& FTL1273L (5′ TATAATCGTTCGTCAACGAG 3′); FTL1273L & LB-TDNA_FTL. The I3 line (*qrt1-2* with two insertions on chromosome 3, FTL1500 CFP at nucleotide position 498916 and FTL1371 DsRed2 at nucleotide position 4319513) as described in Francis et al., [39] was used for EMS mutagenesis as described below.

### Genetic Analysis

Genetic complementation is typically tested by crossing homozygous mutants, but with the *tam* mutants this would introduce a complicating variable since the progeny would be tetraploid. Instead we assessed complementation by crossing heterozygous *tam* plants, selecting double heterozygous progeny with PCR and scoring their phenotype.

A *tam-2* mutant with Col/No-0 polymorphisms was obtained by crossing a heterozygous *tam-2* mutant with a No-0 plant and selfing the F1 generation. We then selected *tam* mutants heterozygous for several Col/No-0 polymorphisms in the F2 generation and crossed them to wild-type Ler plants. The triploid plants obtained were genotyped to infer the genotype of the *tam* 2n gametes.

We obtained *tam-2/spo11-1-3/rec8-3* mutants with Col-0/Ler polymorphisms by crossing a triple heterozygous *tam-2/spo11-1-3/rec8-3* mutant (Col-0) with a wild-type Ler and selfing the F1 progeny. Similarly, plants of interest in the F2 generation were crossed with wild-type No-0 and the triploid progeny were genotyped. The trimorphic (Col-0/Ler/No-0) microsatellite markers used to genotype the *tam-2* (Col-0/No-0) x Ler population and the *tam-2/spo11-1-3/rec8-3* (Col-0/Ler) triple mutant x No-0 F1 population have been described previously [23]. Microsatellite The NGA76 microsatellite was amplified (Tm: 57°C) with the 5′GGAGAAAATGTCACTCTCCACC 3′ and 5′AGGCATGGGAGACATTTACG 3′ primers (35 cycles of 30 sec at 94°C, 30 sec at Tm and 45 sec at 72°C).

### Cytology and Flow Cytometry

Final meiotic products were observed, chromosome spreads generated, and genome sizes were determined as described previously [23]. Pollen fluorescence was analysed as previously described [36]. Images were acquired with a LEICA DM RXA2 epifluorescence microscope using eCFP and DSRed2 filters (Chroma Technologies) and processed with Photoshop 8 (Adobe Systems Inc.).

### Mutagenesis

EMS alleles of the *TAM* locus were generated following the protocol of Weigel and Glazebrook [58]. 0.5 grams of seed were imbibed in 30 ml of sterile water for 4 hrs. and then mutagenized with 0.2% ethyl methane sulfonate for 16 hrs. at room temperature with gentle agitation. Mutagenized seeds were rinsed with 30 ml of sterile water eight times and then dried before planting. Seeds were harvested from individually collected M1 plants. M2 plants were screened for a pollen dyad phenotype (background was *qrt1-2* which produces pollen tetrads).

## Supporting Information

Figure S1 The *tam-2*, *tam-3* and *tam-4* insertions.
(0.03 MB DOC)
