# Supplementary material for: The CYCLIN-A CYCA1;2/TAM Is Required for the Meiosis I to Meiosis II Transition and Cooperates with OSD1 for the Prophase to First Meiotic Division Transition
Source: PLoS Genet. 2010 Jun 17;6(6):e1000989. doi: 10.1371/journal.pgen.1000989 (PMC2887465; doi:10.1371/journal.pgen.1000989)
Supplement: Figure S1 — The tam-2, tam-3 and tam-4 insertions. (0.03 MB DOC) [file pgen.1000989.s001.doc]

ATGC: Genomic sequence

ATGC: T-DNA/Transposon sequence

± xxx distance from *TAM* ATG (bp).

RB3Sail

LB3Sail

*tam-2*

T-DNA

GGTCGGATCTCCCAGCTAACAACTGATAGTTTCCATGGCATATGCT…AGCGTCAATTTGTCCGCAATTTGTTTATCAGCTACTTGGTGTTACCTGCATGATGA

-1796 + 1130

(large genomic deletion)

LBSalk2

*tam-3*

T-DNA

?

CGCAAGTGGAGGATTTCTG…………………………………………..GTTGTCTAAGCGTCAATTTGTTTACACCACAATATATCCTGCTGGTTTCATTAGGTACCA

+1297 +1690

Ds5-2a

Ds3-4

*tam-4*

Ds transposon

TCCGAACTTAGCCAAGACTCGAACTAGGGATGAAAACGGTCGGTA….TACGGGATTTTCCCATCCTACTTTCATCCCTGACTCGAACCTCACTCCGCGATGT

+70 +62

(8 pb genomic duplication)
